# Osmoregulatory disruption and metal bioaccumulation in the estuarine fish *Centropomus parallelus* exposed to settleable atmospheric particulate matter

**DOI:** 10.1007/s00360-026-01698-5

**Published:** 2026-07-27

**Authors:** Anieli Cristina Maraschi, Marco Aurelio Miranda Soares, Viviane Gomes dos Santos, Iara Costa Souza, Magdalena Victoria Monferran, Daniel Alberto Wunderlin, Fabiano Bendhack, Diana Amaral Monteiro, Marisa Narciso Fernandes

**Affiliations:** 1 Departamento de Ciências Fisiológicas (DCF), Centro de Ciências Biológicas e da Saúde (CCBS), Universidade Federal de São Carlos (UFSCar), São Carlos, São Paulo 13565-905 Brazil; 2 Programa Interinstitucional de Pós-Graduação em Ciências Fisiológicas, Universidade Federal de São Carlos/Universidade Estadual Paulista (PIPGCF UFSCar/UNESP), São Carlos, São Paulo 13565-905 Brazil; 3 Centro de Estudos do Mar, Universidade Federal do Paraná, Rua Rio Grande do Norte, 168, Mirasol, Pontal do Paraná, PR Brazil; 4 Center for Research in Clinical Biochemistry and Immunology, Department of Clinical Biochemistry, Faculty of Chemical Sciences, CIBICI – CONICET/UNC), National University of Córdoba, Ciudad Universitaria, 5000 Córdoba, Argentina; 5 ICYTAC, Instituto de Ciencia y Tecnología de Alimentos Córdoba, CONICET and Dpto. Qca. Orgánica, Fac. Cs. Químicas,, Universidad Nacional de Córdoba, Ciudad Universitaria, 5000 Córdoba, Argentina

**Keywords:** Air pollution, Metal, Ionocytes, Gill, Kidney, Osmoregulation

## Abstract

Estuarine fishes experience marked environmental variability and rely on tightly regulated osmoregulatory mechanisms to maintain internal homeostasis. Settleable atmospheric particulate matter (SePM), a complex mixture of metals and metalloids, is an emerging stressor in aquatic systems and may interfere with regulatory processes. This study investigated the effects of SePM exposure on osmoregulatory homeostasis in the estuarine fish *Centropomus parallelus*. Fish were exposed for 96 h to either 0 g.L⁻¹ (control) or 1 g.L⁻¹ SePM, and key physiological endpoints associated with ion and water balance were assessed, including plasma osmolality and Na⁺, Cl⁻, and K⁺ concentrations; Na⁺/K⁺-ATPase (NKA), V-type H^+^-ATPase (VAT), and total ATPase activities in the gills and kidneys; and ionocyte abundance and transporter protein levels in gill ionocytes and renal tubules. Aluminum, iron, vanadium, and chromium levels were accumulated in the gills and kidneys. SePM exposure disrupted osmoregulatory homeostasis, as evidenced by reduced plasma osmolality and decreased Cl^–^ and K^+^ concentrations. NKA activity was upregulated in the gills, accompanied by an increase in the density of NKA-positive ionocytes. Conversely, total ATPase and VAT activities were downregulated in the kidneys, whereas the abundance of NKA, the symporter Na^+^,K^+^,2Cl^−^ (NKCC), and the cystic fibrosis transmembrane conductance regulator (CFTR), a chloride (Cl⁻) channel, remained unchanged in renal tubules. These findings suggest that SePM exposure is associated with tissue-specific modulation of osmoregulatory parameters, including increased branchial Na⁺/K⁺-ATPase activity and NKA-positive ionocyte density, and reduced renal ATPase activities, accompanied by alterations in plasma osmolality and ion concentrations. The physiological disturbances associated with SePM exposure may impair organismal performance and reduce ecological fitness in estuarine fishes inhabiting metal-impacted coastal environments.

## Introduction

Settleable atmospheric particulate matter (SePM), a complex mixture of metals and metalloids, has emerged as a significant environmental concern. SePM originates from metallurgical industries, urban runoff, and other anthropogenic sources (Santos et al. 2017) and contains metals/metalloids such as Al, As, Cu, Fe, Mn, Ti, and Zn, and as well as rare earth elements, which have been identified as emerging contaminants (Santos et al. 2017; Souza et al. 2021a; Monteiro et al. 2023). Upon contact with water, SePM dissociates, releasing metals/metalloids and metallic nanoparticles smaller than 200 nm, which become bioavailable and can be absorbed by aquatic organisms (Souza et al. 2021a).

Estuarine ecosystems, which experience dynamic salinity gradients ranging from freshwater to brackish conditions, are particularly vulnerable to this type of contamination. These ecosystems receive anthropogenic inputs and act as natural barriers, preventing terrestrial inorganic contaminants from reaching the sea (Li et al. 2022). In these environments, metal bioavailability is strongly influenced by fluctuating redox and acid–base conditions (Queiroz et al. 2021), which regulate processes such as precipitation, adsorption, solubilization, and interactions with mineral phases (Li et al. 2022). Salinity plays a key role in these dynamics, influencing the behavior and dissociation of SePM and, consequently, the availability of associated metals and metalloids (Gantayat and Elumalai 2024).

Metal exposure is known to interfere with osmoregulatory processes (McDonald and Wood 1993; Atli and Canli 2007, 2013; Nogueira et al. 2020). In teleosts, the gills represent the primary interface between the internal and external environments, functioning as critical sites for both ion exchange and contaminant uptake (Evans et al. 2005). Together with the kidney and intestine, gills play a central role in osmoregulation and acid-base balance through the activity of specialized ion-transport proteins in ionocytes (Gonzales 2012; Hiroi and McCormick 2012). These cells contain a set of transporters, including the Na⁺/K⁺-ATPase (NKA), the Na⁺-K⁺−2Cl⁻ symporter (NKCC), the cystic fibrosis transmembrane conductance regulator (CFTR), a chloride (Cl⁻) channel, and V-type H^+^-ATPase (VAT) (Marshall 2011; Shih et al. 2023). Among these, NKA and VAT are key drivers of electrochemical gradients that support ion fluxes across branchial epithelia, and their activity and abundance vary with environmental salinity, physiological state, and species (Hiroi and McCormick 2012).

Metals compete with other cations for absorption sites in the gills due to their shared physicochemical properties (Kwong 2024). Essential and nonessential metals may exhibit distinct affinities for membrane carrier proteins, and their toxicity depends on exposure concentration. Among the most abundant SePM-associated elements, Al is known to impair ionoregulatory processes by disrupting branchial ion uptake and inducing structural damage, particularly under low-salinity conditions, and to indirectly affect osmoregulation through oxidative stress and physical alterations of gill surfaces (Wood 1989, 2011). For example, exposure to waterborne Cu, a well-studied metal, increased the activity of NKA in the gills of rainbow trout (*Oncorhynchus mykiss*) (McGeer et al. 2000). Furthermore, Zn exposure inhibits NKA activity in the gill and intestine and calcium (Ca^2+^)-ATPase activity in the muscle of the Nile tilapia *Oreochromis niloticus* (Atli and Canli 2007). Given the vital role of these ion-transport proteins, their modulation or inhibition may disrupt osmoregulatory homeostasis. Therefore, measuring the activity of ion-transport proteins represents a valuable biomarker for assessing the effects of metal contamination, including exposure to SePM-derived mixtures.

The euryhaline and diadromous fat snook *Centropomus parallelus* exhibits strong osmoregulatory capacity, allowing it to thrive in freshwater, brackish water, and marine environments. As a carnivorous species occupying a high trophic level, *C. parallelus* has the potential to biomagnify contaminants, including metals (Souza et al. 2021b). Metals/metalloids have been detected in different tissues of *C. parallelus* following SePM exposure (Monteiro et al. 2023; Maraschi et al. 2025), along with associated biological effects. Previous studies have shown that SePM alters antioxidant defenses in gill, hepatopancreas, and kidney tissues (Monteiro et al. 2023) and induces hematological responses and mutagenic alterations in the blood of *C. parallelus* (Maraschi et al. 2025). The ecological and economic relevance of *C. parallelus*, combined with its frequent occurrence in estuarine environments subjected to anthropogenic contamination, underscores its suitability as a model for investigating the physiological effects of metal exposure in coastal ecosystems (Souza et al. 2013). As an estuarine species, *C. parallelus* relies on highly dynamic osmoregulatory mechanisms to cope with natural salinity fluctuations, making it particularly useful for evaluating contaminant-induced disturbances in ionoregulatory function. However, the integrative effects of multi-metal exposure associated with SePM on fish osmoregulatory physiology remain poorly understood, particularly regarding tissue-specific responses.

Considering the ecological relevance of estuarine fish and the widespread occurrence of metal-rich atmospheric particulate matter in industrialized coastal regions, understanding how exposure to SePM affects osmoregulatory physiology is essential for evaluating its potential impacts on aquatic organisms. In this study, we hypothesized that exposure to SePM-derived metals would be associated with disturbances in systemic osmoregulatory balance in *C. parallelus*, accompanied by tissue-specific modulation of branchial and renal ionoregulatory mechanisms. Specifically, we predicted that SePM exposure would: (i) increase metal accumulation in osmoregulatory tissues; (ii) alter plasma osmolality and ion concentrations; and (iii) modulate ATPase activity and ion-transporter immunoreactivity in gill and kidney ionocytes. To test these predictions, we evaluated metal accumulation, plasma osmoregulatory parameters, enzymatic activities, and the localization of ion-transport proteins in the gills and kidneys of fish exposed to 1 g.L⁻¹ SePM.

## Materials and methods

### SePM sampling

The SePM used in this study was collected on Boi Island, 8 km (as the crow flies) from the Tubarão Industrial Complex, Espírito Santo, Brazil (20°17’03.8 “S, 40°14’24.9 “W). The SePM sampling was conducted in March 2021, with containers positioned on the rooftop of the building, approximately 20 m above ground level. For a more detailed account of the sampling methodology, see Souza et al. (2021a). The sublethal concentration of the tested SePM (1 g.L^− 1^) was selected based on the high deposition rates (10–20 g.m^–2^, 30 days^− 1^) observed in the Metropolitan Region of Vitória (IEMA, 2021). The SePM concentration was selected for its ecological relevance, as it closely matches SePM levels reported in estuarine regions adjacent to steelmaking activity (IEMA 2021; Santos et al. 2017).

The physicochemical characteristics and metal composition of SePM are strongly context-dependent and may vary according to industrial activities, seasonal patterns, meteorological conditions, and atmospheric deposition dynamics. In the present study, the SePM analysis reflected the influence of intense regional steelmaking activity. Elevated levels of Al (6997 µg.g^–1^), Ba (88.1 µg.g^–1^), Ce (18.1 µg.g^–1^), Cr (55.4 µg.g^–1^), Cu (25.5 µg.g^–1^), Fe (106,140 µg.g^–1^), La (9.0 µg.g^–1^), Mn (565 µg.g^–1^), Ni (18.2 µg.g^–1^), Pb (12.6 µg.g^–1^), Rb (2.2 µg.g^–1^), Sn (4.9 µg.g^–1^), Sr (68.5 µg.g^–1^), Ti (708 µg.g^–1^), V (20.4 µg.g^–1^), Y (5.3 µg.g^–1^), Zn (212 µg.g^–1^) and Zr (3.8 µg.g^–1^) was quantified in SePM (Monteiro et al. 2023).

### Experimental procedures

Around 24 specimens of *C. parallelus*, measuring 17 ± 0.3 cm and weighing 80 ± 5.9 g, were collected by reel fishing in the coastal and estuarine regions of the Paraná coast, Brazil (25º35’07.5"S, 48º29’11.4"W). The sampling was authorized under ICMBio/MMA permit #82214-4. All experiments were previously approved by the animal ethics committees of both institutions (CEUA/UFSCAR, No. 2631240523; CEUA/UFPR, No. 12/2022). Fish were transported in aerated 50-L carboys containing water from their respective collections. In the laboratory, individuals were distributed in eight circular tanks (310 L) at a fish density of 1 g.L^–1^ water, as recommended by OECD Technical Standards 203 (2019), containing dilute seawater (salinity 20‰) under constant aeration (> 120 mmHg), temperature (24 ± 2 °C), and a natural photoperiod, during a 15-day acclimation period. All individuals were fed with tilapia fragments daily until 24 h before the start of the exposure.

Two groups, each containing 12 specimens per treatment, were exposed for 96 h in a static system to their respective water conditions under constant aeration (> 120 mmHg): the control group (without SePM) and the SePM-exposed group (with 1 g.L^–1^ SePM). In the exposed tanks, SePM was directly added to the water surface (“sprinkled”) at a concentration of 1 g.L⁻¹ (Monteiro et al. 2023; Maraschi et al. 2024b; 2025). After 48 h of exposure, 50% of the water was renewed, and SePM was reintroduced in the same manner and at the same proportion to maintain the nominal concentration of 1 g L⁻¹ throughout the 96-hour exposure period. Water variables were measured daily in both control and exposed tanks during the exposure period, and there was no difference on water variables between the control and the SePM-group: temperature (22 ± 2 °C; *p* = 0.342), salinity (20‰), dissolved oxygen (7.79 ± 0.18 mg.L^–1^; *p* = 0.314), ammonia (< 0.09 ppm), and pH (7.65 ± 0.16; *p* = 0.152).

After 96 h of exposure, fish were anesthetized with 0.1% benzocaine in water 20‰. The blood sample was drawn from the caudal vein using a #27-5-gauge needle coupled to a 3-mL syringe. The blood samples were centrifuged at 5000 *g* for 10 min to separate the plasma from the cellular fraction, and the plasma was removed for measurement of Na^+^, K^+^, and Cl^–^ concentrations. After collecting blood, the specimens were then euthanized with a higher dose of benzocaine at a lethal concentration of 0.5%, followed by rapid spinal sectioning.

The gills and kidneys were carefully dissected and allocated for specific analyses. The second right gill arch was consistently used for immunofluorescence, ensuring standardized sampling and reliable inter-individual comparisons. The remaining right-side gill arches were designated for metal quantification, while the left-side arches were used for enzymatic activity assays. The entire kidney was sectioned into three segments: the anterior portion was allocated for metal concentration analysis; the middle segment for immunofluorescence; and the posterior segment for ATPase activity assays.

Gill and kidney samples intended for immunofluorescence were immediately fixed in 2% paraformaldehyde (PFA) prepared in phosphate-buffered saline (PBS) for at least 3 h (Bancroft and Gamble 2008; Maraschi et al. 2025a). Tissues designated for enzymatic analysis were stored in 150 mM sucrose, 50 mM imidazole, and 10 mM ethylenediaminetetraacetic acid [EDTA], pH 7.5 (SEI buffer) at − 80 °C (McCormick, 1993; Duarte et al. 2013). The remaining portions, intended for metal quantification, were also stored at − 80 °C until further analysis.

### Metal and metalloid concentration in tissues

The gill and kidney samples were freeze-dried and then macerated and homogenized. A portion of the homogenized tissue sample (0.1 g dry weight) was digested by adding 3 mL of nitric acid (69%), 750 µL of hydrochloric acid (both acids were sub-boiling grade), and 250 µL of hydrogen peroxide. The mixture was incubated for 12 h at 100 °C. The digested samples were filtered through a 0.45-µm nitrocellulose filter (Millipore, Merck, Darmstadt, Germany) in accordance with Agilent inductively coupled plasma–mass spectrometry (ICP-MS) guidelines. A control sample containing all reagents used in the digestion process was also prepared. The samples were stored at 4 °C until analysis.

Using ICP-MS (7500 cx, Agilent Technologies, Santa Clara, CA, USA) equipped with an ASX-100 autosampler (CETAC Technologies, Omaha, NE, USA), according to EPA Method 200.8 (1994), 28 elements (Ag, Al, As, Au, Ba, Bi, Cd, Ce, Cr, Cu, Fe^56^, Fe^57^, Hg^202^, La, Mn, Mo, Nb, Ni, Pb, Rb, Se, Sn, Sr, Ti, V, W, Y, Zn, and Zr) were measured in triplicate. Quality control and quality assurance were assessed using Certified Reference Materials. A solution containing 45 µL of Certipur^®^ Certified Reference Material multi-standard VI (Merck, Darmstadt, Germany) was added to the fish samples before digestion. The remainder of the procedure followed the same steps as the non-spiked samples. The Certified Reference Material recovery was 92.44% ± 13.53% for spiked samples.

### Measurement of osmolality and ionicconcentration

Water and plasma osmolality were measured in undiluted 50-µL aliquots using a freezing-point micro-osmometer (µ-OSMETTE 5004, Precision System, Natick, MA, USA). The water and plasma Na^+^ and K^+^ concentrations were measured in diluted 2000-µL aliquots (1:200, v: v) using a flame photometer (DM-61, Digimed, São Paulo, SP, Brazil). The water and plasma Cl^–^ concentrations were assessed in diluted 10-µL aliquots (1:3, v: v) using a colorimetric kit at 450 nm (Liquid Chloride Kit, Labtest, Lagoa Santa, MG, Brazil). The water Ca^2+^ and magnesium (Mg^2+^) concentrations were measured in 10-µL aliquots (Ca^2+^ 1:4 v/v; Mg^2+^ 1:20 v: v) using a colorimetric kit at 570 and 505 nm, respectively (Calcium or Magnesium Liquiform Kit, Labtest, Brazil).

### NKA, VAT, and total ATPase activities

Gill and kidney samples were homogenized in ice-cold 150 mM sucrose, 50 mM imidazole, 10 mM EDTA, and 2.5 mM sodium deoxycholate, pH 7.5 (SEID buffer) (1:10 w/v), using a homogenizer (TECNAL-TE-103, Piracicaba, SP, Brazil). Homogenates were centrifuged (4 °C, 3,200 *g*) for 7 min, and the supernatant was collected for enzymatic assays. Protein concentration was determined using the Bradford method at 595nm (Bradford 1976).

The Total, NKA, and VAT activities were analyzed according to the basic procedure described by Gibbs and Somero (1989) and Kultz and Somero (1995), as modified by Duarte et al. (2013). Total ATPase activity was initially determined in the absence of inhibitors. Specific NKA activity was calculated as the ouabain-sensitive fraction, whereas VAT activity was calculated as the NEM-sensitive fraction. Thus, the activities of these transport ATPases were estimated from the difference between total ATPase activity and the residual activity measured in the presence of the respective inhibitor. Total ATPase activity was also used as an indicator of the overall ATP-dependent ion-transport capacity in osmoregulatory tissues, providing complementary information on the physiological effects of SePM exposure on membrane-associated transport processes.

The assay is based on adenosine triphosphate (ATP) dephosphorylation and NADPH oxidation in the presence of ouabain, an NKA inhibitor, and on the fraction sensitive to *N*-ethylmaleimide (NEM), a VAT inhibitor. The supernatant (5 µL) and the reaction mixture (195 µL) containing 30 mM imidazole, 45 mM NaCl, 15 mM KCl, 3 mM MgCl_2_, 0.4 mM KCN, 1 mM ATP, 0.2 mM NADH, 3 U/mL pyruvate kinase, 2 U/mL lactate dehydrogenase, 0.1 mM fructose-1,6-diphosphate, and 2 mM phosphoenolpyruvate, were added to the wells of a 96-well microplate. Samples were assayed in quadruplicate: The NKA activity was determined by adding 2 mM ouabain-sensitive fraction, while the VAT activity was determined by adding 2 mM NEM-sensitive fraction, minus the reaction solution without any inhibitor. The rate of NADH oxidation over time was monitored at 340 nm every 30 s for 15 min using an enzyme-linked immunosorbent assay microplate reader (SpectraMax M2, Molecular Devices LLC, San Jose, CA, USA) at room temperature. The Total, NKA, and VAT activities are expressed as µmol ATP mg protein^–1^ h^–1^.

### Immunofluorescence

After fixation in 2% PFA in PBS for at least 3 h, the second gill arches on the right side and the middle segment of the kidney were washed multiple times in PBS to remove excess PFA. The samples were incubated in PBS containing 5% sucrose for 2 h, then in PBS containing 15% sucrose overnight at 4 °C. The gills and kidneys were embedded in a medium cryo-protector (Optimal Cutting Temperature, Tissue-Tek, Sakura, Torrance, CA, USA), immediately frozen in liquid nitrogen, and then stored at − 80 °C, following standard histological procedures (Bancroft and Gamble 2008; McCormick 1995; Maraschi et al. 2025). Non-consecutive sections from the gill and kidney tissue blocks were obtained using a cryostat (CM1850, Leica, Nussloch, BW, Germany) at a thickness of 8–10 μm and mounted on silanized glass slides (StarFrost, Knittel Glass, Braunschweig, Niedersachsen, Germany), with 5 slides prepared per animal. The immunofluorescence procedure used was previously described for fish ion-transport proteins (Hwang et al. 2011; Hiroi and McCormick 2012; Maraschi et al. 2025). The sections were washed with PBS containing 0.1 M glycine and incubated in PBS with 1% bovine serum albumin (BSA) (heat shock fraction, pH 7, ≥ 98%, Sigma-Aldrich) for 1 h to block nonspecific protein binding. Immunolabeling for NKA, NKCC, and CFTR was performed on independent tissue sections using separate antibody staining procedures. The sections were incubated in a wet chamber overnight at 4 °C with the appropriate primary antibody diluted 1:100 in PBS containing 0.1% BSA: mouse anti-NKA (α5 subunit, Development Studies Hybridoma Bank of the University of Iowa, Iowa City, IA, USA), mouse anti-NKCC (T4, Development Studies Hybridoma Bank of the University of Iowa), and mouse anti-CFTR (Cloud-Clone Corp., Katy, TX, USA). After the incubation, the sections were washed with PBS, incubated again in PBS with 1% BSA, and then incubated for 2 h placed the slides in a light tight box at room temperature with the secondary antibody-goat anti-mouse IgG conjugated to the fluorochrome fluorescein (FITC) or rhodamine (TRITC) (Jackson ImmunoResearch, West Grove, PA, USA) - diluted 1:100 in PBS with 0.1% BSA. The sections were washed with PBS and mounted with Fluoromount containing DAPI (Sigma-Aldrich, St. Louis, MO, USA). A coverslip was placed over the sections. The sections were viewed using a BX61 microscope equipped with CellSens Dimension 1.18 software (Olympus Corporation, Tokyo, Japan) for photo documentation. For each animal, 3–5 randomly selected tissue sections were analyzed, with 5 fields per section (*n* = 15–25 fields), to ensure consistent tissue depth across treatments. Images were acquired using a fluorescence microscope (BX61, Olympus Corporation). The density of NKA-, NKCC-, or CFTR-positive ionocytes (cells/mm²) was then quantified. Fluorescence intensity, used to assess the relative abundance of these proteins in kidney ionocytes, was quantified and expressed as arbitrary units per mm^2^. This design ensured both biological replication (five animals per treatment) and technical replication (multiple slides, sections, and images per animal), providing robust and representative measures of ionocyte density across treatments. All image analyses were performed using ImageJ software (Schneider et al. 2012).

### Statistical analysis

PERMANOVA was used to assess the overall multivariate effect of metal concentrations. Subsequently, a two-way ANOVA was conducted separately for each metal to evaluate the effects of SePM exposure and tissue type. Data were transformed when necessary to meet the assumptions of normality and homogeneity of variances. Statistical analyses were not performed for metals whose control levels were below the limit of quantification (LOQ) in that condition or tissue. A p-value of < 0.05 was considered to indicate a statistically significant difference.

Principal coordinate analysis (PCoA) was applied to identify metal accumulation patterns in the gills and kidneys, separately, under control and SePM-exposed conditions. Principal component analysis (PCA) was performed on gill or kidney metal concentrations and osmoregulatory biomarkers under control and SePM-exposed conditions. The expected average contribution criterion was used to identify the variables that contributed most to components 1 and 2. If the contribution of the variables were uniform, then the expected value would be 1/length (variables) (%). The variable with a contribution more remarkable than this cutoff can make a significant contribution to a given component. All univariate and multivariate statistical analyses and visualizations were performed using R version 4.3.3 (R Core Team, 2024). PCoA and PCA were performed using the FactoMineR, factoextra, and ggplot2 packages.

## Results

### Metal and metalloid concentrations in gills and kidneys

Of the 28 quantified metals, 17 were detected in the gills, and 15 were detected in the kidneys. The metal accumulation data for the gills and kidneys are presented in Table 1. Aluminum, Ag, Cd, Cr, Mo, V, and W were determined only in SePM-exposed conditions in gills, and Al, Cr, Mn, Ti, and W were determined in the SePM group in kidneys. Silver, Ni, and Zr were not present in the kidney under any condition. Zirconium was measured only in the gills under the control condition. Molybdenum and V were higher in the kidneys under SePM-exposed conditions than in controls.

**Fig. 1:**
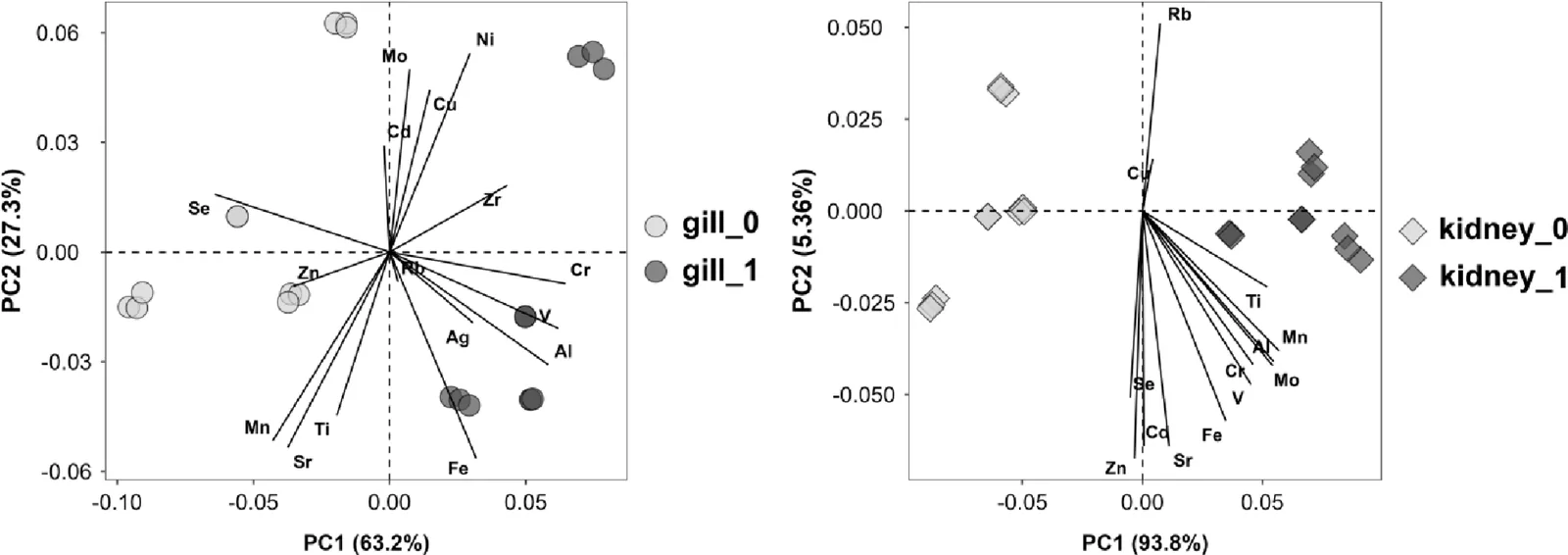
Principal Coordinates Analysis (PCoA) of metal concentrations in the gill (**A**) and kidney (**B**) of *Centropomus parallelus* exposed to control (0 g.L^−1^ SePM; gill_0 and kidney_0) and SePM (1 g.L^−1^ SePM; gill_1 and kidney_1) conditions. Control groups are represented in light grey, while SePM-exposed groups are shown in dark grey

PERMANOVA revealed significant effects of SePM concentration (F = 4.88, *p* = 0.025), tissue (F = 353.88, *p* < 0.0001), and interaction effect (F = 6.26, *p* = 0.018) on the metal concentration. PCoA was performed to examine the distribution of metals in the gills and kidneys separately at the two tested SePM concentrations (0 and 1 g.L^–1^) to further investigate these differences. Multivariate analyses (PCoA) revealed clear tissue-specific patterns of metal accumulation between gills and kidneys, indicating differential acquisition and retention of SePM-derived metals by osmoregulatory organs. In the gills, PCoA explained 90.6% of the total variance (Fig. 1A). PC1, which accounted for 63.2% of the variance, effectively separated the control and SePM-exposed groups. In comparison, PC2 explained 27.3% of the variance within the group. The metals that most strongly contributed to the PCoA variation in gills were Al, Ag, Cr, Fe, V, and Zr. In the kidneys, PCoA explained 99.2% of the total variance (Fig. 1B). PC1, accounting for 93.8%, clearly separated the control group from the SePM-exposed group. In contrast, PC2 explained 5.36% of the variance, accounting for differences in metal accumulation patterns under the same conditions. The metals most significantly associated with SePM exposure in the kidneys were Al, Cr, Fe, Mn, Mo, Ti, and V.

**Table 1:** Metal and metalloid concentrations (µg.g^− 1^) in gill and kidney of estuarine fish *C. parallelus* exposed to 0 g.L^− 1^ (control, CTRL) and 1 g.L^− 1^ SePM (exposed, SePM) for 96 h

Metal	Gills		Kidney		LOQ
	0 g.L^-1^ SePM	1 g.L^-1^ SePM	0 g.L^-1^ SePM	1 g.L^-1^ SePM	
Al	<LOQ	4.23 ± 0.19	<LOQ	64.97 ± 5.88	2.19
Ag	<LOQ	0.05 ± 0.01	<LOQ	<LOQ	0.03
Cd	<LOQ	0.01 ± 0.00	0.10 ± 0.02	0.11 ± 0.01	0.03
Cr	<LOQ	5.33 ± 0.45	<LOQ	20.87 ± 4.40	0.03
Cu	2.66 ± 0.09	2.61 ± 0.09	6.81 ± 0.16	7.58 ± 0.66	0.65
Fe	129.24 ± 7.31^a^	205.52 ± 18.22^b^	454.08 ± 45.07^A^	806.26 ± 36.89^B^	1.00
Mn	2.95 ± 0.49	1.64 ± 0.31	<LOQ	6.08 ± 0.41	0.03
Mo	<LOQ	0.07 ± 0.01	0.13 ± 0.02	1.05 ± 0.09^*^	0.07
Ni	0.26 ± 0.05	0.34 ± 0.03	<LOQ	<LOQ	0.01
Rb	3.80 ± 0.06^a^	3.77 ± 0.14^a^	4.18 ± 0.22^A^	4.36 ± 0.10^A^	0.01
Se	3.77 ± 0.03^a^	2.92 ± 0.07^a^	12.66 ± 0.65^A^	11.77 ± 0.29^A^	0.36
Sr	37.89 ± 7.46^a^	26.70 ± 4.98^b^	18.26 ± 0.87^A^	20.69 ± 0.49^A^	0.15
Ti	2.54 ± 0.37	2.37 ± 0.30	<LOQ	6.34 ± 0.85	1.01
V	<LOQ	0.26 ± 0.01	0.26 ± 0.03	1.70 ± 0.23*	0.04
W	<LOQ	0.02 ± 0.00	<LOQ	0.08 ± 0.02	0.01
Zn	67.23 ± 3.03^a^	63.24 ± 1.38^a^	2281.42 ± 265.34^A^	2168.60 ± 48.11^A^	0.44
Zr	0.07 ± 0.02	<LOQ	<LOQ	<LOQ	0.11

Different letters indicate significant differences (*p* < 0.05). Lowercase and uppercase letters indicate differences between tissues. Values different from the control are highlighted with * *LOQ*: Limit of quantification of each metal analyzed in the ICP-MS

### Osmolality and ionic concentration of the external medium and plasma

The osmolality of the external medium (586 ± 3.08 mOsm kg H₂O^–1^; *p* = 0.465) and pH (7.65 ± 0.16; *p* = 0.152) remained stable regardless of SePM exposure. Similarly, the concentrations of major ions in external water, Na^+^ (325 ± 4.8 mM), K^+^ (7.1 ± 0.2 mM), Cl^–^ (365 ± 14 mM), Ca^2+^ (10.7 ± 0.95 mg/dL), and Mg^2+^ (60.1 ± 3 mg/dL), showed no significant difference between the groups (0.141 < *p* < 0.643).

**Fig. 2:**
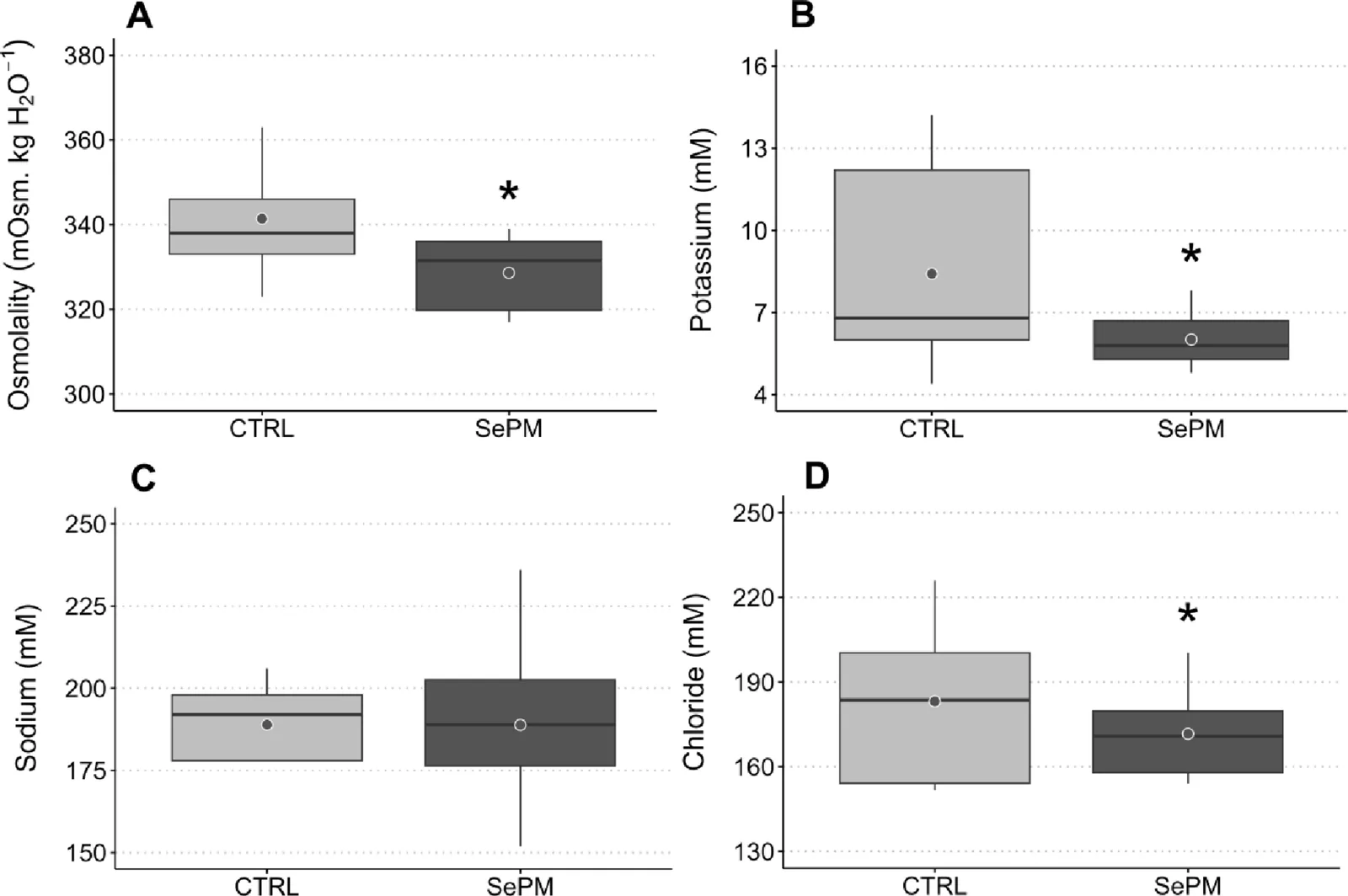
Plasma osmolality (mOsm.kg H_2_O^-1^) (**A**), potassium (mM) (**B**), sodium (mM) (**C**), and chloride (mM) (**D**) concentration in the *C. parallelus* exposed to control (CTRL) and to 1 g.L^-1^ SePM (SePM) conditions. The osmolality and ion concentration of the external medium correspond to salinity 20‰: 600 mOsm.kg H_2_O^-1^, [Na^+^] ~280 mM; [Cl^-^]~320 mM; and [K^+^] ~6 mM. Boxplots represent the distribution of data for each experimental group, where the median line indicates the median of the data, the bottom of the box represents the first quartile (Q1), and the top of the box represents the third quartile (Q3). The black dot indicates the mean value for each group.The * indicates the difference between the groups

Exposure to SePM at 1 g.L^–1^ decreased the plasma osmolality (*p* = 0.01) (Fig. 2A) and the K^+^ (*p* = 0.04) (Fig. 2B) and Cl^–^ concentrations (*p* = 0.04) (Fig. 2D) of *C. parallelus*. The Na^+^ concentration remained unaltered (*p* = 0.99) (Fig. 2C). Despite these changes, plasma osmolality and ion concentrations remained lower than those in the external medium in both the control and SePM-exposed groups. Specifically, the K^+^ concentration decreased from 8.42 ± 0.92 mM in the control group to 6.02 ± 0.28 mM in the SePM-exposed group, reducing the hyper-regulatory capacity relative to the external medium.

### ATPase activities

**Fig. 3:**
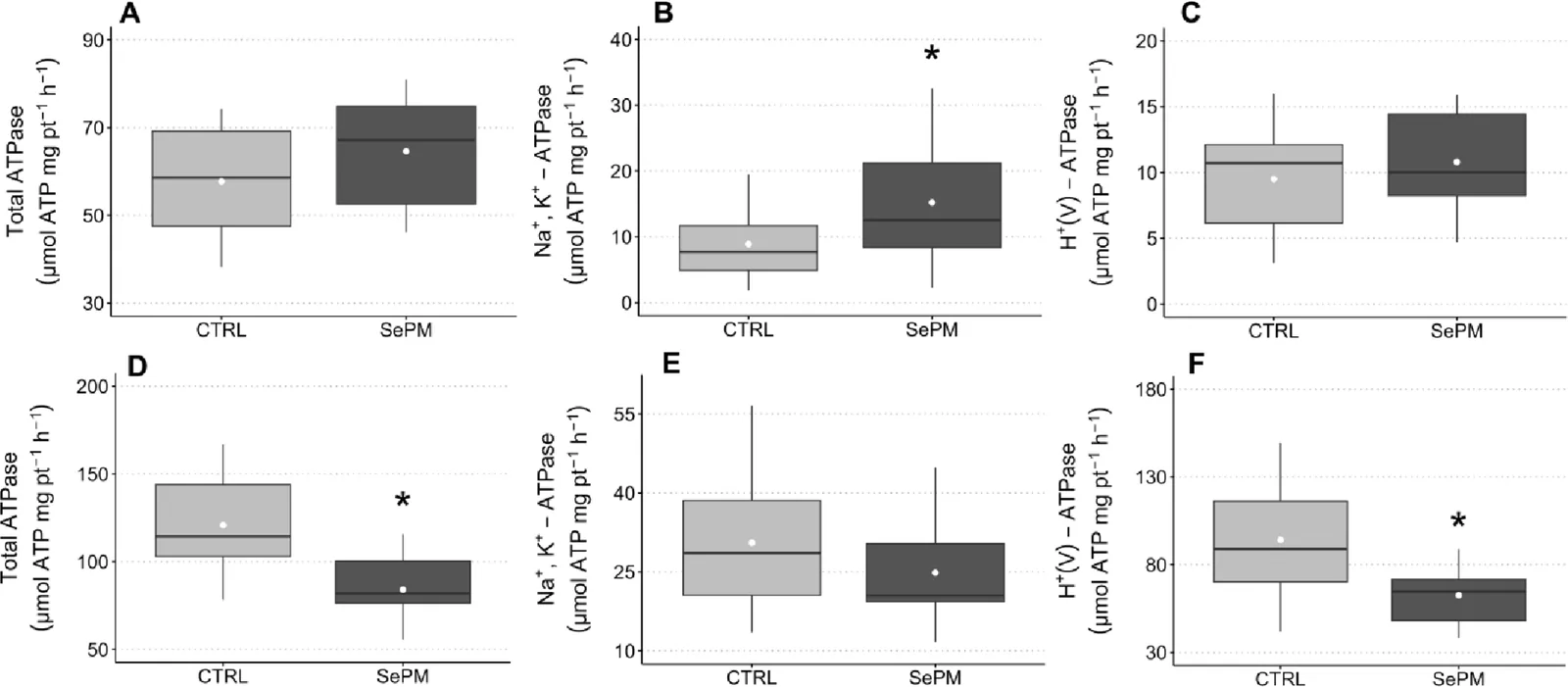
Total ATPase, Na^+^/K^+^-ATPase (NKA), and V-type H+-ATPase (VAT) activities (µmol ATP mg protein^− 1^ h^− 1^) in the gill (**A**, **B**, and **C**) and kidney (**D**, **E**, and **F**) of *C. parallelus* exposed to control (CTRL) to 1 g.L^− 1^ SePM (SePM) conditions. Boxplots represent the distribution of data for each experimental group, where the median line indicates the median of the data, the bottom of the box represents the first quartile (Q1), and the top of the box represents the third quartile (Q3). The black dot indicates the mean value for each group. The * indicates the difference between the groups

In the gills of *C. parallelus* exposed to SePM, NKA activity increased by 94.5% compared with the control group (*p* = 0.049; Fig. 3B), while the total ATPase and VAT activities showed no significant changes (*p* = 0.120 and *p* = 0.245, respectively; Fig. 3A and C). Conversely, in the kidneys, the total ATPase and VAT activities decreased by 30.5% (*p* = 0.008) and 33.5% (*p* = 0.026), respectively, in the SePM-exposed group compared with the control group (Fig. 3D and F), while the NKA activity did not differ between the groups (*p* = 0.334; Fig. 3E).

### NKA, NKCC, and CFTR immunolocalization

#### Gills

**Fig. 4:**
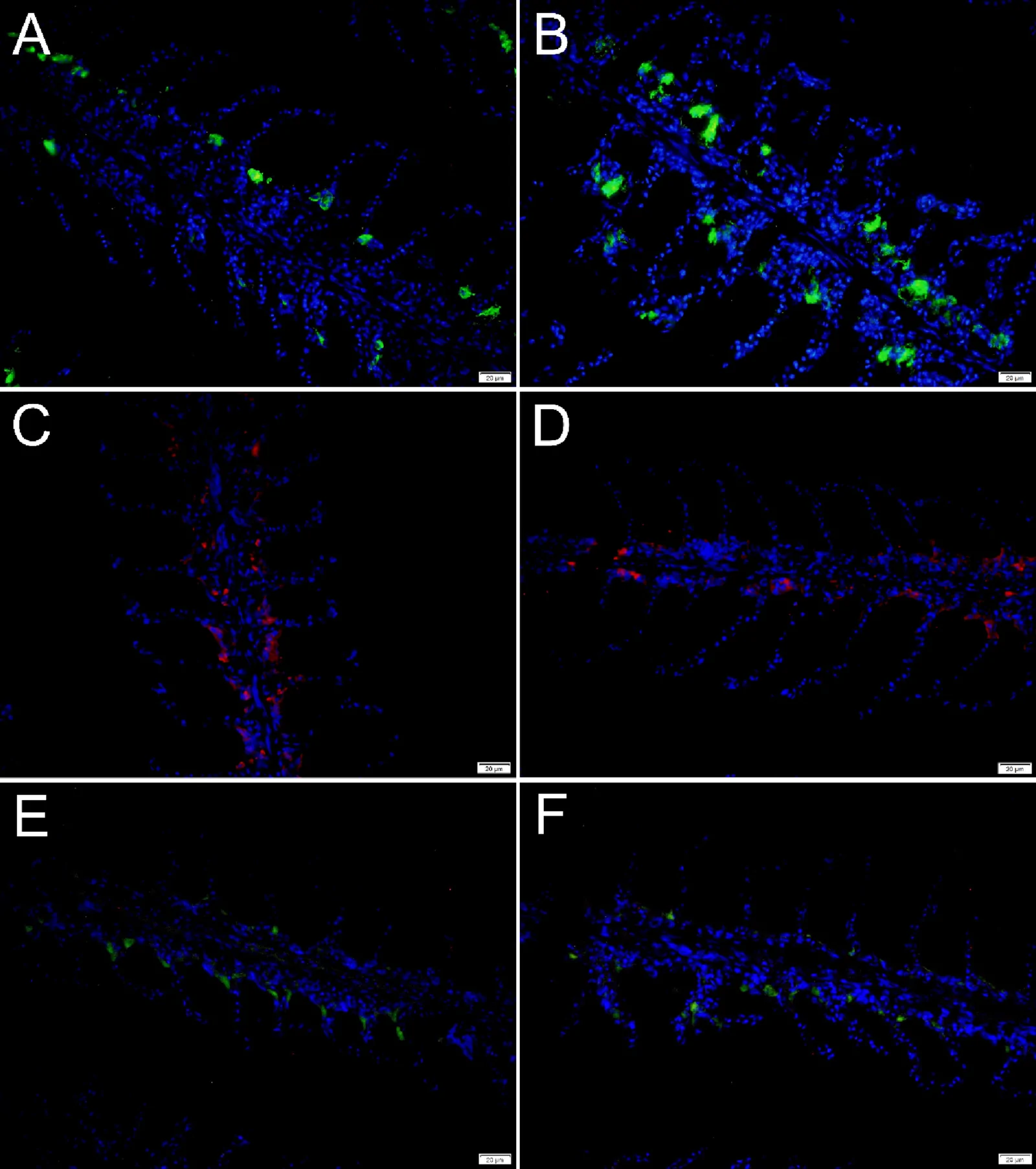
Gill immunofluorescence of *C. parallelus* under control (**A, C, E**) (0 g.L⁻¹ SePM) and exposed (**B, D, F**) (1 g.L⁻¹ SePM) conditions. Na⁺/K⁺-ATPase (NKA) (**A, B**) and Cl^−^ channel (CFTR) (**E, F**) are indicated in green, and Na^+^, K^+^, 2Cl^−^ (NKCC) symporter is indicated in red. Cell nuclei are stained blue with DAPI. Scale bars: 20 μm

Immunofluorescence revealed the presence of NKA in the ionocytes of the gill lamellae, particularly at the intersection between the primary and secondary lamellae (Fig. 4A and B). NKA fluorescence was absent in the secondary lamellae. The number of NKA-positive ionocytes per area of gill lamellae was higher in the SePM-exposed group (865.57 ± 24.53 cells/mm^2^) compared with the control group (652.08 ± 34.49 cells/mm^2^) (*p* < 0.05).

NKCC immunofluorescence was mainly localized to the gill primary lamellae, but it was also found in the secondary lamellae (Fig. 4C and D). The number of NKCC-positive ionocytes per area of gill lamellae was not different between the control (512.39 ± 65.89 cells/mm^2^) and SePM-exposed (567.94 ± 62.11 cells/mm^2^) groups (*p* = 0.555). CFTR immunofluorescence was evident in both primary and secondary lamellae of the gill (Fig. 4E and 4F). The number of CFTR-positive ionocytes per area of gill lamellae was not different between the control (774.93 ± 120.27 cells/mm^2^) and SePM-exposed (647.85 ± 101.15 cells/mm^2^) groups (*p* = 0.437).

#### Kidneys

**Fig. 5:**
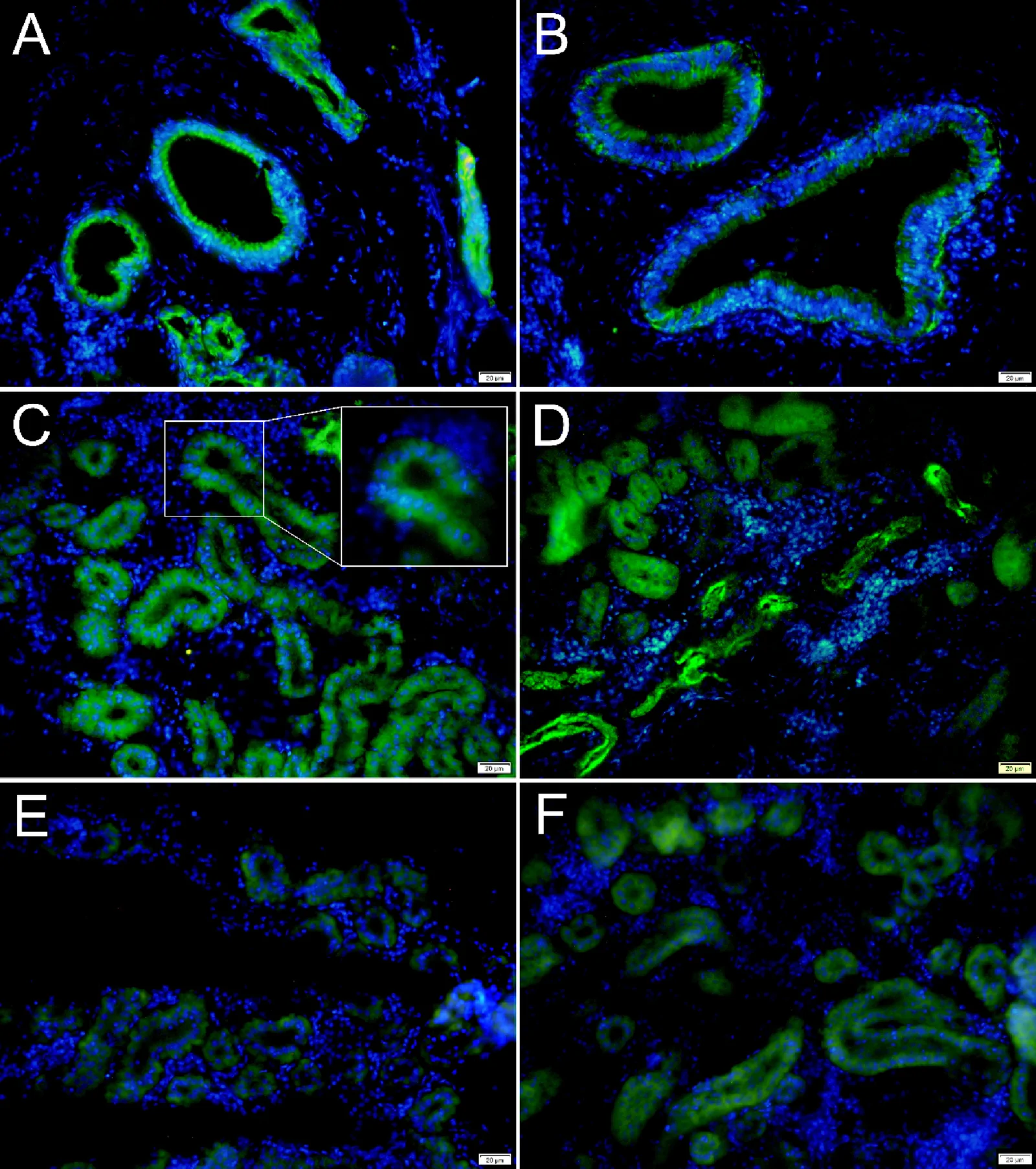
Kidney immunofluorescence of *C. parallelus* under control (**A, C, E**) (0 g.L⁻¹ SePM) and exposed (**B, D, F**) (1 g.L⁻¹ SePM) conditions for 96 h. Na⁺/K⁺-ATPase (NKA), Na^+^, K^+^, 2Cl^-^ (NKCC), and Cl^-^ channel (CFTR) are indicated in green. Cell nuclei are stained blue with DAPI. Scale bars: 20 μm

Immunofluorescence revealed the presence of NKA, NKCC, and CFTR in renal tubule ionocytes. The fluorescence intensity (arbitrary units) per area of renal tubule did not differ between the control and SePM-exposed groups for NKA (28491 ± 3152.2 and 29082 ± 4968.8 au/mm^2^, respectively; *p* = 0.920), NKCC (36346 ± 6632.1 and 38941 ± 7373.6 au/mm^2^, respectively; *p* = 0.811), and CFTR (38043 ± 6621.9 and 42136 ± 6999.2 au/mm^2^, respectively; *p* = 0.692) (Fig. 5).

### Multivariate analysis

**Fig. 6:**
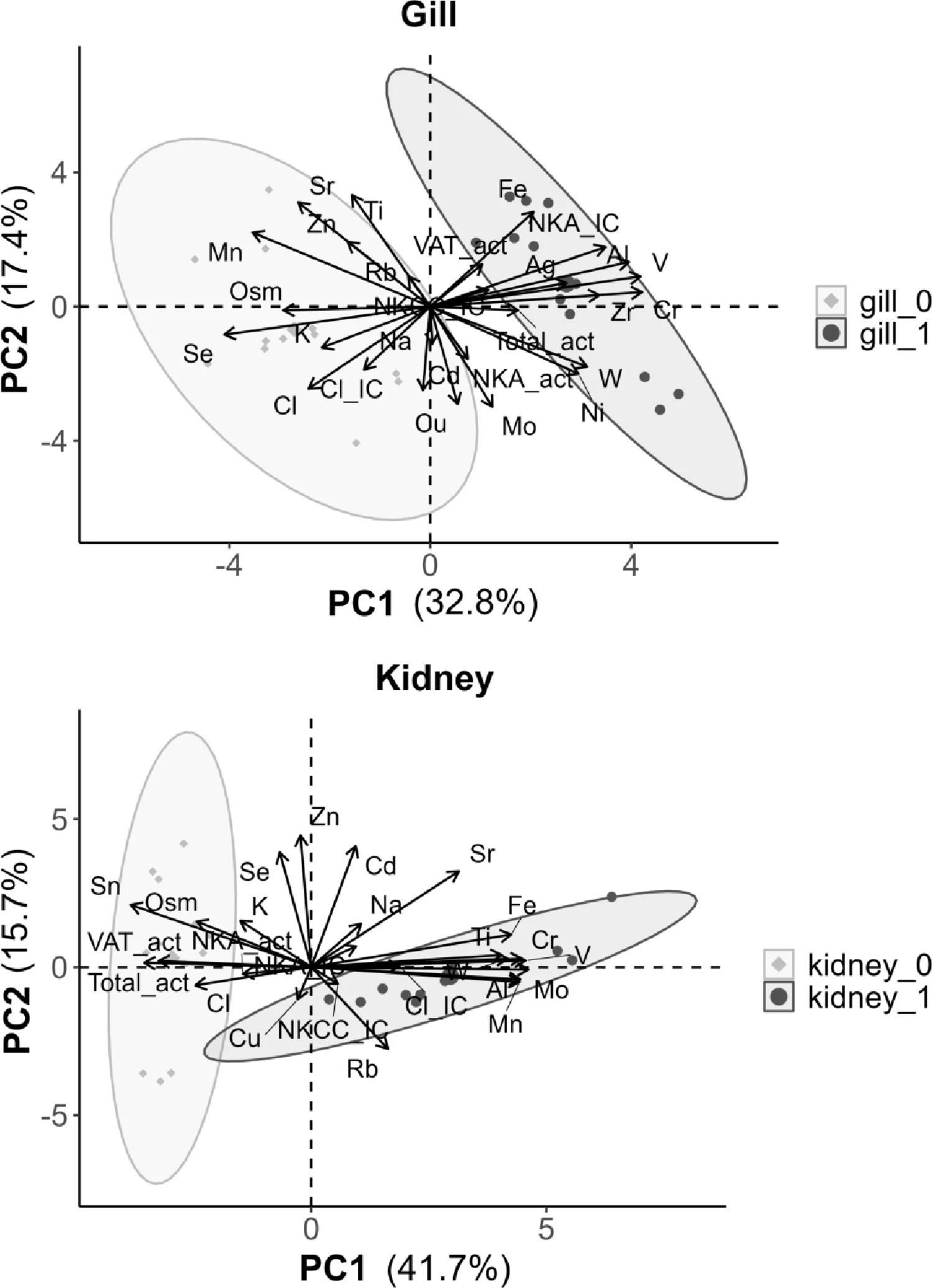
Principal components analysis (PCA) of metal concentrations and biomarker responses in the gill and kidney of *C. parallelus* exposed to control (0 g.L^−1^ SePM; gill_0 and kidney_0) and SePM (1 g.L^−1^ SePM; gill_1 and kidney_1) conditions. Control groups are represented in light grey, while SePM-exposed groups are shown in dark grey

PCA, including all metals detected in tissues and osmoregulatory biomarkers, was performed separately for the gills and kidneys (Fig. 6) to evaluate potential associations between tissue-specific metal accumulation and physiological responses. In the gills, PCA explained 50.2% of the total variance. PC1 explained 32.8% of the opposite clustering between the control (gill_0) and SePM-exposed (gill_1) groups, while PC2 explained 17.4% of the variance. The most representative metals in the SePM-exposed group (> cutoff) were Al, V, Cr, Fe, Zr, Ni, and Ag. Among the biomarkers, the number of NKA-positive ionocytes was particularly representative. In the kidneys, PCA explained 57.4% of the total variance. PC1 accounted for 41.7% of the variance in the opposite clustering between the control (kidney_0) and SePM-exposed (kidney_1) groups, while PC2 accounted for 15.7%. The most representative metals in the SePM-exposed group (> cutoff) were Al, Cr, Fe, Mn, Mo, Sr, Ti, V, and W, along with total ATPase and VAT activities. Overall, the PCA supported the association between tissue-specific metal accumulation patterns and distinct osmoregulatory responses in gills and kidneys following SePM exposure.

## Discussion

Estuarine fishes rely on finely tuned osmoregulatory mechanisms to cope with pronounced physicochemical variability. In the present study, exposure to settleable atmospheric particulate matter (SePM) was associated with disturbances in osmoregulatory homeostasis in *Centropomus parallelus*, as evidenced by reduced plasma osmolality and ion concentrations (Fig. 2), increased NKA activity and ionocyte density in the gills (Figs. 3 and 4), and decreased ATPase activities in the kidney (Fig. 3). These findings support our first hypothesis that SePM-derived metals accumulate in osmoregulatory tissues and alter systemic osmoregulatory balance, while also indicating tissue-specific physiological disturbances consistent with our second and third hypotheses.

While the effects of individual metals on fish osmoregulation are relatively well described (Grosell et al. 2002; Grosell and Wood 2002; Carmo et al. 2018, 2019), the integrative effects of multi-metal exposure remain poorly understood. The present results provide evidence that combined exposure to SePM-derived metals leads to coordinated yet tissue-specific osmoregulatory responses. It is important to note that under semi-static experimental conditions, factors such as water chemistry, redox status, and particle settling may influence metal dissolution dynamics and colloidal stability. Thus, the observed accumulation patterns likely reflect both environmental availability and physicochemical transformations occurring during exposure.

The exact metal profile of SePM may vary regionally according to industrial activities, seasonality, meteorological conditions, and atmospheric deposition dynamics. Nevertheless, multimetal atmospheric particulate contamination associated with industrial and steelmaking activities has been widely reported in different regions worldwide, including settleable particulate matter in highly industrialized areas (Negral et al. 2021), as well as fine atmospheric particulate fractions from industrial regions in Australia (Mohiuddin et al. 2014), China (Zhang et al. 2020), and Europe (Mbengue et al. 2017). Although these studies evaluated different atmospheric particle fractions, they collectively demonstrate the recurrent occurrence of metal-enriched atmospheric particulates in industrialized environments.

### Tissue-specific metal bioaccumulation following SePM exposure

Exposure to SePM for 96 h increased metal accumulation in both gills and kidneys of *C. parallelus* (Table 1), with a compositional pattern that closely matched the SePM metal signature. In the gills, Al, Ag, Cr, Fe, V, and Zr were the main contributors to the multivariate pattern (PCoA; Fig. 1). At the same time, the kidneys showed increased concentrations of Al, Cr, Fe, Mn, Mo, Ti, V, and W. This pattern suggests that metal uptake is influenced not only by environmental availability but also by tissue-specific physiological functions.

The PCoA approach was useful for identifying tissue-specific patterns of metal accumulation associated with SePM exposure, revealing differential metal acquisition between gills and kidneys. This multivariate analysis provided an integrative assessment of tissue-specific metal accumulation, supporting the interpretation of differential physiological responses between osmoregulatory organs. Tissue-specific metal accumulation is influenced by the rates of metal uptake and clearance among organs (Luoma and Rainbow 2005). Gills are the primary interface between fish and the external environment and may facilitate the adsorption and uptake of dissolved metals via ion transport pathways and epithelial binding sites (Evans et al. 2005). In contrast, kidneys are directly involved in filtration, ion regulation, and excretory processes, which may contribute to the accumulation and retention of circulating metals and metal complexes (Marshall and Grosell 2005). Additionally, differences in metal speciation, affinity for transport proteins, and competition with physiologically relevant ions may also influence tissue-specific accumulation patterns. For example, Ag accumulation in gills may be associated with its interaction with Na⁺ transport pathways and high affinity for branchial epithelial surfaces (Marshall et al. 2002; Wood et al. 2010), whereas the greater renal accumulation of elements such as Mo and V may reflect systemic circulation and renal handling of oxyanion forms (Eisler 1989; Rehder 2015). Chromium distribution may additionally depend on metal speciation and differential tissue affinity (Velma et al. 2009).

In the present study, Al and Fe concentrations increased by 93% and 59%, respectively, in the gills, and by 2867% and 78% in the kidneys of SePM-exposed fish (Table 1). Similar patterns of metal accumulation following SePM exposure have been reported across different taxa (Souza et al. 2021b; Fortes et al. 2023; Monteiro et al. 2023; Nobre et al. 2024; Maraschi et al. 2024b, c), supporting the consistency of the present findings. The SePM exposure for 96 h increased Al by 425% and Fe by 511% in the gills of freshwater *O. niloticus* (Maraschi et al. 2024c); Al by 613% and Fe by 3046% in the muscle of bullfrog tadpoles *Aquarana catesbeiana* (Fernandes et al. 2024); Al by 619% and Fe by 29.6% in the whole-blood of American bullfrog tadpoles *Lithobates catesbeianus* (Costa et al. 2024); and by Al by 8590% and Fe by 484% in whole-body of fiddler crab *Minuca rapax* (Maraschi et al. 2024b).

Under external conditions of salinity 20‰ and pH 7.65 ± 0.16, Al and Fe tend to form oxyhydroxide colloids that precipitate rapidly, resulting in low dissolved concentrations. Despite this, both metals accumulated substantially in gill and kidney tissues in the present study (Table 1), indicating effective bioavailability. This accumulation may be explained by microenvironmental processes at the gill surface, where local acidification promotes the redissolution of metal colloids (Playle and Wood 1989), allowing interaction with ion-transport sites and potentially contributing to the osmoregulatory disturbances observed in this study.

Exposure to Al causes an ionic imbalance in freshwater fish, reducing osmolality and the concentrations of Na^+^, Cl^–^, and K^+^ (Dussault et al. 2001; Camargo et al. 2009; Grassie et al. 2013). These effects are likely associated with Al-induced alterations in NKA activity, changes in the distribution and abundance of branchial ionocytes, and the activation of stress responses (Camargo et al. 2009). High Fe concentrations in different species exposed to SePM are also correlated to biological damage (Maraschi et al. 2024b, c). Sayadi et al. (2020) demonstrated that sublethal exposure to iron oxide nanoparticles and Fe salts results in marked Fe accumulation in the gills and intestines of the blackfish, *Capoeta fusca*, and consequently disrupts tissue structure (and likely function). In zebrafish (*Danio rerio*), dietary Fe induces transient changes in the messenger RNA (mRNA) levels of various metal transporters and the expression of the epithelial Ca^2+^ channels (Chandrapalan and Kwong 2020). However, little is known about the effects of Fe on fish osmoregulatory mechanisms.

In addition to Al and Fe, other metals, such as Cr, V, Mn, Ag, Mo, and W, accumulated in osmoregulatory tissues. These elements may interfere with ion transport through multiple mechanisms, including competition with physiological ions (Atli and Canli 2013; Nogueira et al. 2020; Kwong 2024), oxidative stress (Briffa et al. 2020; Hernroth et al. 2020; Kumar et al. 2023), and disruption of membrane proteins (Briffa et al. 2020; Horng et al. 2023). However, given the multimetal nature of SePM, isolating the contribution of individual elements remains challenging (Briffa et al. 2020; Nogueira et al. 2020).

### Physiological responses

Osmoregulatory mechanisms are widely recognized as sensitive indicators of physiological stress in response to contaminant exposure (Saglam et al. 2013). In *C. parallelus*, SePM exposure resulted in reduced plasma osmolality and decreased Cl⁻ and K⁺ concentrations (Fig. 2), indicating disruption of ionic homeostasis. These changes likely reflect impaired ion uptake and/or increased ion loss across epithelia, consistent with functional disturbances in osmoregulatory organs, although the precise molecular pathways underlying these effects were not directly investigated in the present study. Reduced plasma Cl⁻ and K⁺ concentrations can alter membrane electrochemical gradients, promoting hyperpolarization and impairing excitability-dependent physiological processes. Disturbances in ion balance can affect neuromuscular and cardiovascular function (Le et al. 2026), ultimately compromising swimming performance, predator avoidance, and foraging efficiency. Comparable systemic adjustments have been reported in other organisms exposed to SePM, including increased cardiac output in *O. niloticus* (Adorno et al. 2023) and reduced behavioral responses in fiddler crabs (Maraschi et al. 2024c), suggesting an allostatic adjustment associated with the maintenance of functional homeostasis. Maintaining osmotic and ionic balance under these conditions may impose a substantial energetic cost. The reallocation of metabolic resources to support compensatory osmoregulatory mechanisms may reduce the energy available for growth and reproduction.

The maintenance of ionic balance in fish involves the coordinated activity of NKA, located in the basolateral membrane of ionocytes, along with other enzymes, such as VAT, and ion-transport proteins, including the NKCC symporter and the CFTR channel (Marshall 2011; Shih et al. 2023). Together, these components form a complex branchial and renal transepithelial exchange system. As described in the previous section, this osmoregulatory machinery is inherently sensitive to metal exposure. Moreover, structural damage to the gills caused by metal toxicity compromises the functionality of key transporters in the gill epithelium (e.g., NKA, VAT, Ca^2+^-ATPase, and Mg^2+^-ATPase), ultimately disrupting osmoregulatory homeostasis in the organism (Wood et al. 2010; Briffa et al. 2020; Horng et al. 2023).

In our study, SePM exposure increased NKA activity in the gills (Fig. 2) and was accompanied by a higher density of NKA-positive ionocytes (Fig. 3), suggesting a compensatory response to maintain ion balance. This upregulation may result from increased ionocytes differentiation or modulation of enzyme activity. Nevertheless, it could also stem from changes in enzyme kinetics resulting from the activation or inhibition of regulatory molecules (Leone et al. 2023). For example, NKA activity in the gills of the blue crab *Callinectes danae* can be stimulated by cobalt (Co^2+^) in the absence of Mg^2+^. Although Co^2+^ exhibits a 4.5-fold greater affinity for NKA than Mg^2+^, the latter competitively displaces Co^2+^ from the Mg^2+^-binding site in a concentration-dependent manner (Leone et al. 2023). Given that many metals in the complex SePM share properties similar to those of Co^2+^, their presence may contribute to diverse and variable physiological responses.

In contrast, the kidneys exhibited reduced total ATPase and VAT activities following SePM exposure (Fig. 3), indicating impaired renal ion-transport capacity. The decline in enzymatic activity may be attributed to the high affinity of metals for sulfhydryl (–SH) groups in enzymes, leading to oxidation, disruption of hydrogen bonding, and alterations in protein conformation (Briffa et al. 2020). Additionally, membrane damage, disturbances in ion homeostasis (Uren Webster et al. 2013), and depletion of the energy budget (Maraschi et al. 2025c) caused by metal exposure may further contribute to enzymatic inhibition. Accordingly, the complexity of multi-metal exposure may obscure clear mechanistic patterns, as different metals can exert opposing effects on enzyme kinetics and transporter regulation.

The absence of changes in NKCC- or CFTR-positive ionocyte density or fluorescence intensity does not necessarily indicate preserved function (Figs. 4 and 5). The regulation of these transporters often involves complex phosphorylation mechanisms, which cannot be inferred solely from changes in protein abundance (Delpire and Gagnon 2018), and their activity is closely linked to ATPase-driven processes. Therefore, reductions in ATPase activity (Fig. 3) may have downstream effects on ion transport even in the absence of detectable changes in protein expression.

Taken together, the increase in branchial NKA activity and ionocyte density, alongside the reduction in renal ATPase activities, indicate a coordinated, tissue-specific adjustment of osmoregulatory mechanisms in *C. parallelus* exposed to SePM. This differential modulation aligns with the observed decreases in plasma osmolality and Cl⁻ and K⁺ concentrations (Fig. 2), suggesting a coordinated physiological adjustment in which branchial ion uptake is enhanced while renal ion regulation is compromised. Overall, the present findings support the hypothesis that SePM-derived multi-metal exposure is associated with tissue-specific disturbances in osmoregulatory homeostasis. The coordinated increase in branchial ion-transport capacity and the reduction in renal ATPase activity are consistent with an integrative physiological adjustment to maintain internal homeostasis under exposure to metal-rich particulates.

PCA enabled the evaluation of biological associations between accumulated metals and osmoregulatory biomarkers, helping identify which metals were more strongly associated with specific physiological alterations in each tissue. An important aspect of the present study is that the observed physiological responses likely reflect the combined effects of multiple metals rather than the action of a single element. In environmentally relevant mixtures such as SePM, metals may interact through additive, synergistic, or antagonistic mechanisms, potentially altering ion transport processes and enzyme regulation in ways that differ from those observed with single-metal exposures (Briffa et al. 2020). Moreover, different metals may exert contrasting effects on ATPase activity and membrane transport depending on their chemical properties, affinity for transport proteins, and competition with physiologically relevant ions. Therefore, the osmoregulatory disturbances observed in *C. parallelus* probably result from the integrated effects of complex multi-metal exposure, which may partially explain the tissue-specific physiological responses identified in the present study.

**Fig. 7:**
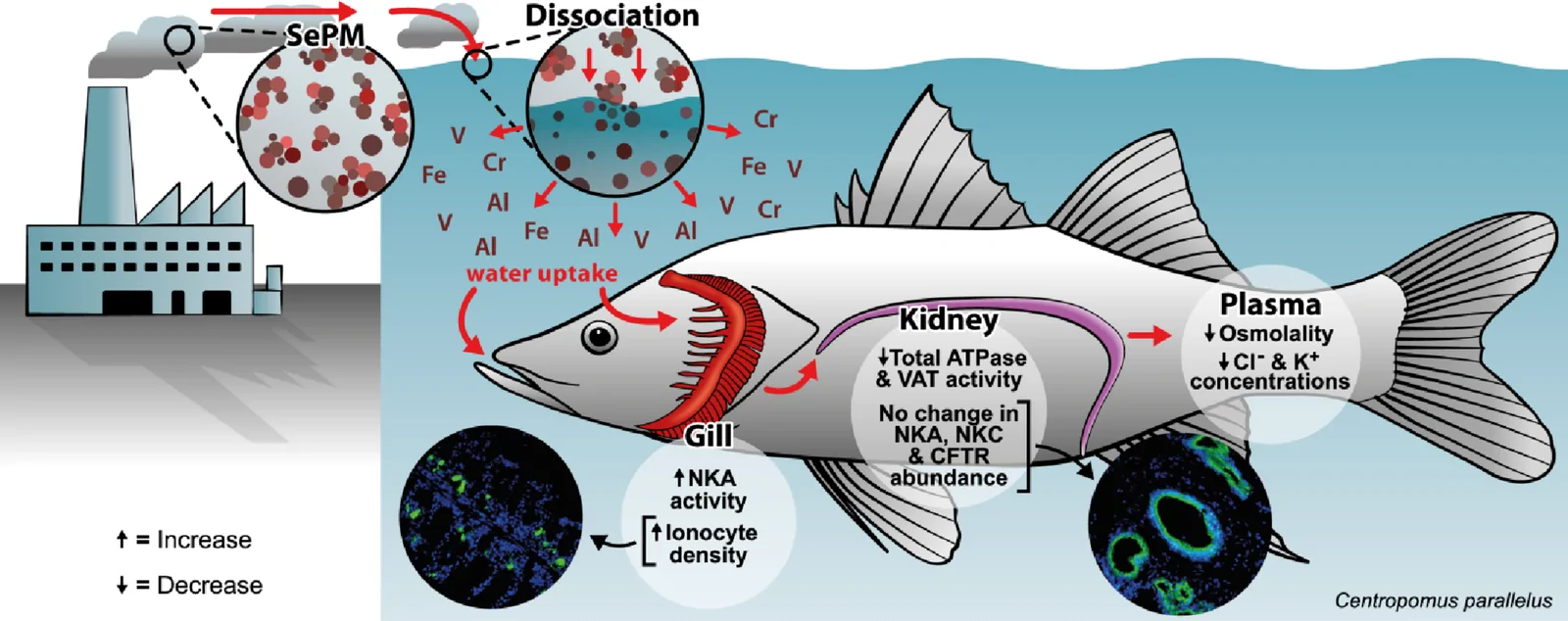
Conceptual model illustrating the associations observed between SePM exposure, tissue-specific metal accumulation, alterations in ion transport proteins and ATPase activities, and changes in plasma ionic balance in *Centropomus parallelus*. The model summarizes potential physiological pathways that may contribute to osmoregulatory disturbance following short-term exposure to environmentally derived particulate matter

Based on the present findings, we propose an integrative model summarizing the potential pathways by which SePM-derived metals may affect osmoregulatory physiology in *C. parallelus* (Fig. 7). Future studies should aim to disentangle the relative contributions of individual metals within complex mixtures and investigate the molecular pathways underlying transporter regulation, providing deeper insights into the effects of metal exposure on transporters that do not function primarily through ATP-dependent mechanisms. Such approaches will be essential for improving our understanding of the ecological risks associated with SePM in coastal ecosystems.

## Conclusion

The association between metal accumulation and physiological responses provides essential insights into the effects of SePM on osmoregulatory homeostasis and allostatic regulation in *C. parallelus.* Multivariate analyses revealed strong relationships between the metals most accumulated in the gills and kidneys, particularly Al, V, Cr, and Fe, and alterations in osmoregulatory balance. These findings support a physiologically consistent association between SePM exposure and disturbances in osmoregulatory function, characterized by increased branchial Na⁺/K⁺-ATPase activity and ionocyte density, concomitant with reduced total ATPase and V-type H^+^-ATPase activities in the kidneys. This tissue-specific modulation suggests differential physiological adjustments between osmoregulatory organs during SePM exposure. Such disturbances are likely to impair key biological functions and reduce ecological performance. Overall, these results underscore the environmental risks of SePM contamination in aquatic environments and emphasize the importance of accounting for its complex metal composition when assessing impacts on aquatic biota.

## Data Availability

Data will be available on request.
